# An Updated Overview on the Regulation of Seed Germination

**DOI:** 10.3390/plants9060703

**Published:** 2020-06-01

**Authors:** Gerardo Carrera-Castaño, Julián Calleja-Cabrera, Mónica Pernas, Luis Gómez, Luis Oñate-Sánchez

**Affiliations:** Centro de Biotecnología y Genómica de Plantas, (Universidad Politécnica de Madrid-Instituto Nacional de Investigación y Tecnología Agraria y Alimentaria), Campus de Montegancedo, Pozuelo de Alarcón, 28223 Madrid, Spain; gerardo.carrera@upm.es (G.C.-C.); julian.calleja@upm.es (J.C.-C.); pernas.monica@inia.es (M.P.); luis.gomez@upm.es (L.G.)

**Keywords:** ABA/GA, hormone signaling and dynamics, transcription factors, environmental signals, seed dormancy and germination, epigenetics, post-transcriptional regulation, spatio-temporal regulation

## Abstract

The ability of a seed to germinate and establish a plant at the right time of year is of vital importance from an ecological and economical point of view. Due to the fragility of these early growth stages, their swiftness and robustness will impact later developmental stages and crop yield. These traits are modulated by a continuous interaction between the genetic makeup of the plant and the environment from seed production to germination stages. In this review, we have summarized the established knowledge on the control of seed germination from a molecular and a genetic perspective. This serves as a “backbone” to integrate the latest developments in the field. These include the link of germination to events occurring in the mother plant influenced by the environment, the impact of changes in the chromatin landscape, the discovery of new players and new insights related to well-known master regulators. Finally, results from recent studies on hormone transport, signaling, and biophysical and mechanical tissue properties are underscoring the relevance of tissue-specific regulation and the interplay of signals in this crucial developmental process.

## 1. General Introduction

Seed production and germination are intimately connected and closely linked to the survival and dispersal of plant species. The main role of the seed is to protect the embryo and sense environmental information to couple germination with seasons compatible with the completion of the plant life cycle. Germination encompasses the events from imbibition to radicle protrusion through the seed coverings. In the field, the spatial pattern of seed dispersal depends on the habitat of the mother plant as well as on the fruit and seed morphology. In addition, the temporal distribution of germination mainly depends on the interaction between the environment and the plant’s genetic makeup, which conditions both dormancy and germination potential. For instance, it is known that seeds developed in plants exposed to low temperatures will have higher dormancy levels and the opposite when supplied with nitrate. Physiological dormancy, the most common type [1,2], provides seeds with valuable advantages. First, it maximizes dispersion, thus reducing competition for resources between the offspring and the mother plant. Second, it halts germination in the wrong season, even if short spells of favorable conditions occur. After reaching maturity, seeds undergo a process called after-ripening (AR) characterized by a gradual reduction in water content and dormancy level, whose speed depends on the relationship of seed moisture content and temperature during dry storage. At this point, non-dormant seeds can retrieve the dormancy program upon encountering inadequate conditions (secondary dormancy) or, if conditions are adequate, proceed to germination. In this case, the intake of water or imbibition by the non-dormant seed triggers different biochemical, metabolic and physiological processes, such as the resumption of respiratory activity, energy production, activation of repair mechanisms, protein biosynthesis from both stored and newly synthesized mRNAs and reserve mobilization. These events fuel the elongation of the embryonic axis and the weakening of the embryo surrounding tissues, leading to rupture of the seed coat (testa rupture), embryo radicle protrusion (germination *sensu stricto*) and seedling establishment.

To comprehend the processes taking place in the seed, it is necessary to have a deep understanding of the molecular and biochemical mechanisms that regulate them. This review intends to illustrate the key issues in a comprehensive and readable form, keeping a reasonable extension. Nevertheless, supplementary tables with a compilation of selected reviews that expand on specific aspects of seed biology (Table S1) and a list of complete gene names and their abbreviations (Table S2) have also been included. We have focused this review on *Arabidopsis thaliana* (Arabidopsis) although relevant findings in other plant species have been included. In the first part, we will describe the molecular players and networks controlling these processes, and their links to environmental and hormonal cues. In the second part we will review these processes from a genetic and physiological perspective.

## 2. Regulatory Layers Controlling Seed Germination

### 2.1. Hormone Metabolism and Signaling

Germination depends on the physiological state (dormancy) of the seed, which is partly caused by the interaction between the plant genotype and a wide spectrum of environmental factors, such as temperature, soil moisture, light and nutrient availability. This is mainly achieved through regulation of the metabolism and signaling of gibberellins (GAs) and abscisic acid (ABA), two phytohormones with antagonistic roles. Their spatio-temporal balance plays a pivotal role in seed biology by favoring dormancy over germination when the ABA/GA ratio is high, and the opposite when it is low [3]. In fact, the first dormancy- and germination-associated loci identified in Arabidopsis mutants included genes involved in GA and ABA biosynthesis, perception and signaling [3,4,5,6,7,8]. Bioactive GAs are formed in terminal reactions catalyzed by GA20ox and GA3ox oxidases. Deactivation of GAs by GA2ox oxidase and transcriptional feedback loops are also important features in GA homeostasis [9,10]. In particular, the GA3ox1 and GA3ox2 enzymes for biosynthesis, and GA2ox2 for catabolism, stand out for their key role in GA signaling during germination [11,12,13]. There are three main components involved in early perception and signaling by GAs: the GID1 (GA receptor) and the GID2 (F-box) proteins are positive signaling regulators [14,15,16], while DELLA proteins act as negative regulators [17,18]. The presence of GAs triggers the interaction of its receptor GID1 with DELLAs through their N-terminal domain (DELLA domain) and the formation of a ubiquitination complex via interaction with GID2. This interaction induces the proteasome-mediated degradation of DELLAs [19,20,21,22]. DELLA mutant versions lacking the DELLA domain are resistant to degradation and confer GA insensitivity [23,24]. DELLAs negatively regulate GA signaling through protein-protein interactions with several transcriptional regulators [19,20,25,26,27,28]. In Arabidopsis there are five DELLAs, of which RGL2 has a major role in regulating germination, since its loss-of-function mutants are able to restore germination of GA-deficient seeds [29,30]. Regarding the two Arabidopsis GID2 proteins, SLY1 seems to have the dominant role in germination, since SNE/SLY2 overexpression does not produce a decrease in RGL2 protein levels [31]. For the three *GID1* genes found in Arabidopsis, a double mutant *gid1ac* had to be obtained to observe defects on germination [32], whereas GID1b has an ABA-independent role in AR [33]. 

The key enzymes involved in ABA biosynthesis are NCED dioxygenases, while CYP707A monooxygenases are central to ABA catabolism through 8’-hydroxylation [34,35]. In particular, the loss of function of two ABA biosynthetic enzymes, NCED6 and NCED9, results in dormancy reduction [36] while mutation of the *CYP707A2* gene decreases germination potential [37,38,39]. As in the case of GAs, three main components are involved in early ABA perception and signaling: the ABA receptor (PYR/PYL/RCAR) proteins and the SnRK2 protein kinases, which are positive regulators of the pathway, and PP2Cs protein phosphatases as negative regulators. ABA-bound receptors are able to bind and inhibit PP2Cs which, in turn, allow phosphorylation and activation of SnRK2s [35,40,41,42]. Activated SnRK2s phosphorylate downstream targets, such as the ABI5 and other members of the AREB/ABFs bZIP transcription factors (TF) family [43], among others, to activate the ABA response in plants [35]. In the seed, responses to ABA and related physiological processes are mainly under the control of ABI5 together with two other non-bZIP TFs, ABI3 (B3 family TF) and ABI4 (ERF family TF) [35,42]. Among these regulators, ABI3 is the one acting upstream ABI5 and ABI4 and is essential for *ABI5* expression in germination arrest [44], whereas ABI4 acts as a repressor of lipid breakdown in the embryo [45]. Both TFs are positive regulators of the expression of *ABI5* during seed germination [44,46]. Furthermore, ABI5 activates its own expression by binding to its own promoter [47].

### 2.2. Hormone Dynamics and Transport

The spatio-temporal action of plant hormones is crucial for proper development and germination [48,49,50,51]. Compelling evidence of temporal and tissue-specific regulation of hormone metabolism and signaling in seeds have been obtained, and recent results are improving our view on hormone transport in this organ [11,45,52,53,54,55,56,57,58,59,60,61,62]. For instance, the release of ABA from the endosperm into the embryo controls its growth and maintains its dormancy in dry seeds, a role that requires RGL2 function [63]. It has also been found that temperature shifts alter the spatial distribution of GAs and ABA in dormant embryos, suggesting that crosstalk mediated by hormone transport occurs between cell types in the embryonic axis [61]. Four AtABCG transporters expressed specifically in seed tissues were found to act in concert to correctly deliver ABA to control seed germination: AtABCG25 and AtABCG31 export ABA from the endosperm to the embryo, whereas AtABCG30 and AtABCG40 import ABA into the embryo from the endosperm. Consequently, it has been proposed that radicle extension and subsequent embryonic growth are suppressed by the coordinated activity of multiple ABA transporters expressed in different seed tissues [64]. 

The AtSWEET13 and AtSWEET14 transporter proteins were found to mediate cellular GA uptake when expressed in yeast and oocytes [65]. The *sweet13*/*sweet14* double mutant exhibits altered long distance transport of exogenously applied GAs and their wild type (WT) versions are required for proper development of seeds and seedlings. SWEET family proteins were initially identified as sugar transporters and specific members of the family are involved in seed filling [66,67,68]. However, their role as GA transporters during seed development may not be so relevant for germination. In fact, GAs stored in dry seeds are not, or not sufficiently, transmitted to the offspring to successfully complete germination under permissive conditions, since de novo synthesis of GAs is required at this stage [69]. An intriguing observation is that the seeds produced by *sweet13/sweet14* plants were larger than WT seeds but less sensitive to inhibition of germination by ABA or paclobutrazol (PAC, a GA biosynthesis inhibitor). If the SWEET proteins promoted GA influx into seed tissues, their loss of function would be expected to reduce seed size and increase sensitivity to ABA and PAC-mediated inhibition of germination [65].

The *NPF3* gene encodes a protein targeted to the plant cell membrane where it functions as a GA influx transporter [70,71]. NPF3 belongs to the *NPF* gene family previously reported to encode nitrate or peptide transporters, some of which are also able to transport hormones [72,73]. Whereas *NPF3* is expressed during seed development, *npf3* mutants do not show altered germination under standard conditions, maybe due to genetic redundancy. Yet, these mutants showed a reduced germination response to GAs under nitrogen-limiting conditions [70,71]. NPF3 is also an ABA transporter in vitro and its expression is upregulated by low nitrogen, light and ABA and downregulated by high GA levels [70,71]. Interestingly, enhanced expression of *NPF3* has been associated with a greater propensity to break dormancy. This effect has been proposed to be related to altered ABA/GA balance due to enhanced capacity for GA intake [61]. These findings suggest a role for NPF3 as a negative regulator of dormancy subjected to GA negative feedback. Another member of this family previously known as a low-affinity nitrate transporter (NPF4.6/AIT1/NRT1.2) [74], also mediates ABA uptake in yeast and insect cells. Compared with WT plants, the *npf4.6/ait1/nrt1.2* mutant was less sensitive to exogenously applied ABA during seed germination and/or post-germination growth [75]. The fact that NPF proteins may act in vivo as dual transporters of nitrate and hormones, along with the dependence of their mutants on nitrogen availability to show altered germination responses, suggests the existence of molecular crosstalk that adapts germination to the nutrient environment. NLP6 TF is a NIN-like (NLP) protein that was found to control gene expression in response to the nitrate signal in vegetative stages [76]. NLP6 as well as other NLP family proteins bind a specific *cis*-element (NRE) in the promoters of nitrate-responsive genes and activate their expression. Genes involved in nitrate assimilation (NIA, NIR1) and transport (NRT1.1, NRT2.1) as well as regulatory genes of both processes are targets of NLP6 [76,77]. When nitrate is fed to the mother plant, seeds have reduced levels of dormancy, partly because ABA levels are reduced [78]. Not surprisingly, the *CYP707A2* gene is induced by nitrate and, when mutated, seeds are less sensitive to nitrate-induced dormancy release [79]. Recently, a direct link between nitrate and ABA metabolism was found by revealing the role of NLP8, a TF that reduces ABA levels in a nitrate-dependent manner by directly binding to the promoter of *CYP707A2* [80]. 

In summary, it is now clear that seeds respond to the nutrient environment perception by linking transport of nitrogen to hormones. By using specific nitrogen-dependent regulators, seeds can also modify gene networks and hormonal balance to modulate dormancy and germination. Additional information on this regulation can be found at the end of the next section.

### 2.3. Environmental Influence of Transcriptional Regulation: Expanding the Regulatory Breadth of Known/Classic TFs

**Figure 1:**
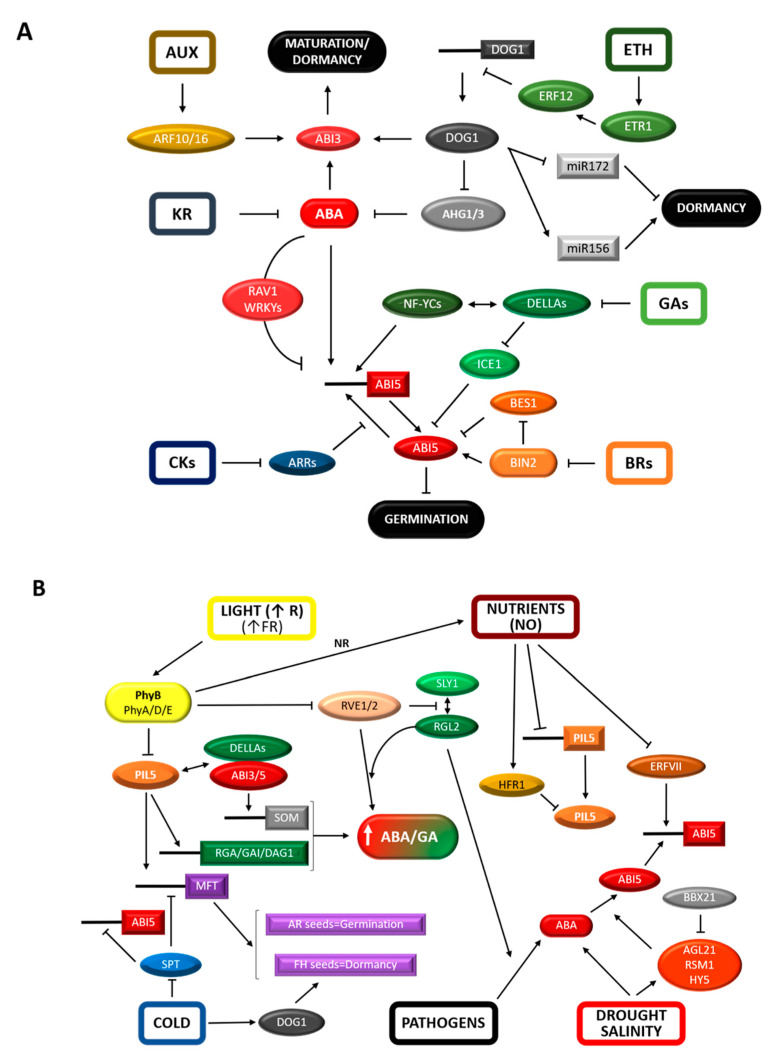
Interplay between core components of molecular mechanisms controlling seed dormancy and germination with hormones and environmental signals. (**A**) Hormonal and molecular regulatory networks involved in dormancy and germination. DOG1 increases abscisic acid (ABA) sensitivity through sequestration of PP2Cs (AHG1/3) and genetically interacts with *ABI3* to ensure ABA signaling during seed maturation and the establishment of dormancy. *DOG1* expression is regulated by ethylene (ETH) signaling through the ETR1/ERF12 pathway and has an impact on dormancy release through the control of two antagonistic miRNAs. Other hormones such as auxin (AUX) by the ARF10/16 pathway and karrikins (KR) have a role in dormancy by altering ABA content or signaling. ABI5 plays a key role in ABA signaling to repress germination. *ABI5* expression is upregulated under conditions unfavorable for germination by several TFs (ABA-related TFs or NF-YC3/4/9). Negative feedback by RAV1 and WRKY18/40/60 or conditions promoting germination counteract this upregulation. Also, other hormonal signaling pathways (gibberellins, GAs; brassinosteroids, BRs; cytokinins, CKs) interfere with ABI5-mediated transcription or stability through several regulatory proteins (DELLAs, ICE1, BES1, BIN2 or ARR4/5/6). (**B**) Effects of environmental factors on the regulation of seed dormancy and germination. PIL5 represses germination in the absence of light. It increases the ABA/GA balance partly through direct upregulation of *SOM*, *DELLAs (RGA/GAI)* and *DAG1* transcription. ABI3/5 and DELLAs also participate in the upregulation of *SOM* gene transcription. Upon light perception, PIL5 activity is counteracted by different mechanisms mostly mediated by phytochromes (PhyB): increased degradation and reduced transcription in response to higher NO levels and reduced function through HFR1 competitive interaction. NO has also an effect on the stability of class VII ERFs, mediating their degradation and thus reducing *ABI5* expression. In addition, activated Phys also reduce *DELLA* (*RGL2*) expression and increases its degradation by reducing the expression of circadian genes (RVE1/2). DOG1 integrates temperature cues to regulate dormancy release in fresh-harvested seeds. In dry seeds, SPT negatively regulates germination in the absence of low temperatures. SPT activates *ABI5* and represses *MFT* expression. MFT induces dormancy in fresh seeds but promotes germination in AR seeds, and it is a convergence point between PIL5 and SPT regulation. Pathogen perception triggers DELLA-dependent and GA-independent *ABI5* expression to block germination in anticipation of potential seedling damage. Drought and salinity stimulate ABA biosynthesis and induce *ABI5* expression, a response mediated by HY5, RSM1 and AGL21 and counteracted by BBX21.

The antagonism between GAs and ABA fine tunes seed germination to environmental conditions. The regulation exerted by the ABI5 TF (Figure 1) is one of the central nodes of this antagonism [81]. Expression of *ABI5* is stimulated by ABA, water stress and high salinity, a response that relies on three TFs that bind to and activate its promoter, namely HY5/HYH, RSM1 and AGL21. Loss-of-function mutants of these genes decrease sensitivity to ABA, salinity and water stress during seed germination [82,83,84]. Although HY5 and BBX21 TFs are positive regulators of photomorphogenesis, their interaction counteracts HY5 upregulation of the *ABI5* promoter. This mechanism is also used by BBX21 to interfere with ABI5 upregulation of its own promoter [85]. Similarly, VQ18 and VQ26, which belong to a recently identified family of plant-specific transcriptional regulators [86], bind to ABI5 to interfere with its transcriptional activity [87]. Reduction of ABI5 function is also controlled through negative feedback by the ABI5-induced AHT1, a BTB/POZ-domain containing protein which is a potential receptor for the proteasome CRL3 complex [88]. 

Additional proteins participate in the negative regulation of ABA signaling in germinating seeds: (1) the RAV1 TF represses *ABI5* expression when is phosphorylated by SnRK2 kinases, in sharp contrast with the positive effect that these kinases have on the ABA pathway through phosphorylation of some early signaling components [89]; (2) a loss-of-function mutant of an MDN1 domain-containing protein (SAG) shows higher levels of *ABI5* and *ABI3* mRNAs in the presence of ABA [90]; (3) three WRKY-domain TFs (WRKY18, WRKY40, and WRKY60) repress *ABI4* and *ABI5* expression by direct binding to their gene promoters [91,92]; (4) *ABI5* mRNA levels increase in the *AtMyb7* TF loss-of-function mutant [93]. 

A clear example of ABA and GA signaling integration during seed germination is provided by the interaction between the RGL2 DELLA protein and three NF-YC TF homologs (NF-YC3, NF-YC4 and NF-YC9). This module directly upregulates *ABI5* gene expression through specific binding to CCAAT elements in the *ABI5* promoter [94]. Another example is the ICE1 TF, previously known for its positive role in cold-induced responses [95]. It has been reported that ICE1 binds to ABI5 and interferes with its transcriptional activity. In addition, DELLAs interact with ICE1 to repress its effect on ABI5 [96]. 

The central role of the ABA/GA balance in seed biology is integrated with other hormones, such as brassinosteroids (BRs) and cytokinins (CKs). The binding of ABI5 to the BIN2 kinase, a key repressor of BR signaling, promotes its phosphorylation and stability [97]. On the other hand, the positive regulator of BR signaling BES1 TF interacts with ABI5 to interfere with its transcriptional activity [98]. These findings are in line with the role proposed for BRs in reducing ABA sensitivity during germination [99]. The A-type ARR TF proteins are primary targets of cytokinin signaling and negative feedback regulators of the pathway. Specific members of this family (ARR4, ARR5 and ARR6) were found to interact with ABI5 and reciprocally downregulate their transcription [100]. After germination *sensu stricto*, cytokinin promotes the proteasomal degradation of ABI5 and regulates cotyledon greening, mainly via ARR12 [101]. Pathogens also influence germination. A biotic compound released by *Pseudomonas aeruginosa* is perceived by the seed and triggers DELLA-dependent and GA-independent *ABI5* expression to block germination in anticipation of potential seedling damage [102]. In summary, current knowledge suggests that ABI5 is an important hub protein, regulated at different levels, where several hormonal and environmental signals converge. The roles of additional hormones are reviewed elsewhere in the text.

Light is one of the most important environmental cues and molecular links have been found between seed responses to adverse light conditions and ABA-mediated repression of seed germination. In AR seeds and under unfavorable conditions, ABI5, DELLAs and ABI3 form a complex that directly activates the transcription of the SOM TF. This factor then negatively regulates seed germination by increasing the ABA/GA balance [103,104,105]. ABI3 also interacts with PIL5/PIF1 TF to collaboratively activate *SOM* expression by binding to its promoter [104]. PIL5 represents a link between hormonal signaling and light-regulated germination [106]. Phytochromes (Phy) are a class of plant photoreceptors that enter the nucleus upon light activation, recruiting PIL5 away from its DNA-binding sites and triggering fast PIL5 degradation, and hence seed germination [107,108,109]. PIL5 degradation under light conditions is mediated by the COP1–SPA1–CUL4 E3 ubiquitin ligase (CUL4 complex) [110], where SPA1 is necessary for PIL5 phosphorylation and subsequent ubiquitination by the CUL4 complex [111]. In addition to CUL4 complex-mediated degradation, PIL5 is polyubiquitinated and subsequently degraded by the KELCH F-box protein CTG10 in association with an E3 ubiquitin ligase (SCF-complex) [112,113]. When light influx is low, Phy-mediated signaling is not enough to remove PIL5 [108]. Under these conditions, the HFR1 TF is able to effectively sequester the remaining PIL5, so its transcriptional activity is suppressed to ensure rapid germination [114]. In darkness, Phys are inactive and the COP10–DET1–DDB1–CUL4 E3 ligase complex targets and degrades HFR1 by using DET1 and COP10 as substrate receptors. Moreover, DET1 and COP10 directly interact with PIL5 to prevent its 26S proteasome-mediated degradation and favor its stability [115]. PIL5 then directly binds to and activates the *GAI* and *RGA* promoters [116]. It also represses GA biosynthesis (*GA3ox1* and *GA3ox2*) and activates GA catabolism (*GA2ox*) partly through promoter binding and activation of *SOM* and *DAG1* TFs transcription [103,117,118,119]. PIL5 typically binds to G-box elements in target promoters [116,120], but it can target additional binding sites depending on its interaction with other TFs [121]. Another aspect of the germination response to light is that far-red light inactivates PhyB mainly in the endosperm, initially preventing germination through PIL5 stabilization. Simultaneous activation of PhyA in the embryo leads to a slow destabilization of PIL5, accompanied by a weakening of ABA-dependent responses and eventually to germination in the absence of testa rupture [122,123,124]. PhyA-mediated germination has been interpreted as the last opportunity for seeds to develop a seedling despite the presence of unfavorable light conditions (e.g., far-red-enriched canopy light). The expression of several genes involved in this response has been found to be independent of PIL5, suggesting that PhyA action is regulated by additional TFs [125,126]. Interestingly, the PIF8/UNE10 TF inhibits phyA-induced seed germination without affecting phyB-mediated responses, suggesting a role as an attenuator of the photomorphogenic development under long term far-red conditions [127]. Besides PIL5 and PIF8, PIF6/PIL2 TF also plays a role in seeds. It is expressed strongly during seed development and its loss increases primary dormancy [128]. Less studied phytochromes such as PhyD and PhyE, stimulate germination under high far-red light fluence, probably by promoting PhyA action [129,130]. PhyD also has a role in relieving secondary dormancy in seeds exposed to high temperature through PIL5 removal [131]. Oppositely, PhyC negatively regulates seed responses to light, a function that depends on other phytochromes and the formation of heterodimers between them [130,132,133]. However, these phytochromes play minor roles compared with PhyB [130]. 

Besides light, other environmental factors such as the presence of nitrate or low temperatures stimulate the biosynthesis of GAs and promote germination of mature seeds [11,78]. In the absence of low temperatures the light-stable SPT TF suppresses the expression of *GA3ox* and represses germination. SPT is degraded upon cold treatment thus removing the repression of germination [134]. In the absence of light and cold stimuli PIL5 and SPT block germination in a complementary manner [135]. In addition, SPT binds the *ABI5* and *MFT* (phosphatidylethanolamine-binding protein) promoters, activating *ABI5* and repressing *MFT* expression, respectively [136]. Interestingly, while MFT induces dormancy in freshly-harvested seeds, it promotes germination in AR seeds through negative feedback on the ABA signaling pathway [136,137]. An interaction between light and cold signaling has also been observed in the control of germination. It has been proposed that MFT functions as a convergence point between light and cold regulation since its expression is promoted by PIL5 under far red light and, in the absence of cold stimulus, repressed by SPT under red light. This is in agreement with the observation that PIL5 downregulates *SPT* expression, an additional checkpoint to block germination in the dark [135].

Nitrate is the major nitrogen source for most plant species and plants sense the nutritional environment through nitric oxide (NO) synthesis from nitrate. NO has been indeed proposed as the key signaling element mediating nitrogen responses which promotes dormancy breaking and germination [138,139]. PhyB activation has been linked to the stimulation of nitrate reductase (NR) activity and NO accumulation [140]. In turn, NO signals downregulate the transcription of *PIL5* and stabilize HFR1 protein to intensify the HFR1-PIL5 interaction, which counteracts the inhibitory effect of PIL5 on its target genes [140]. NO also reduces ABA sensitivity at least by promoting CYP707A2 synthesis, probably through enhanced transcription mediated by NLP8 [80], thus leading to ABA degradation [141]. Molecular evidence of the crosstalk between NO and ABA signaling was provided by showing that *ABI5* expression is reduced through the NO-mediated activation of the N-end rule pathway targeting class VII ERFs TFs for degradation [142]. Another study found that NO controls ABI5 protein stability through S-nitrosylation, which triggers ABI5 ubiquitination by the KEG E3 ligase and degradation by the 26S proteasome [143]. Interestingly, ABA antagonizes this effect by promoting KEG degradation [144]. In addition, NO produces S-nitrosylation and inactivation of SnRK2 kinases required for phosphorylation and activation of ABI5 [145]. 

### 2.4. Germination Control by the Epigenome

Several studies have revealed that epigenomic mechanisms are able to modulate the expression of genes related to dormancy, maturation and germination. Specific chromatin modifiers and remodelers have been shown to promote seed dormancy or germination by enhancing and/or repressing the expression of specific gene subsets (Table 1). 

A number of chromatin modifications which alter transcription initiation and elongation positively regulate the expression of dormancy-related genes. The *HUB1/RDO4* E3 ubiquitin ligase gene, like its homolog *HUB2*, is required for H2B histone monoubiquitination and expression of dormancy-related genes [146]. The H2B monoubiquitination is a chromatin modification associated with promoting transcription initiation and early elongation events [147]. *RDO2* encodes a transcription elongation factor (TFIIS) and, similarly to *hub1*, the *rdo2* single mutant has reduced dormancy and share with it about 30% of downregulated genes [148,149]. The reduction of DOG1 levels, a central regulator of dormancy (discussed below), partly explains the phenotype of the *rdo2* mutant [150]. Arabidopsis ATXR7 is a H3K4 methyltransferase that, when mutated, produce similar effects on dormancy as those described for *hub1* and *rdo2* mutants [149]. Interestingly, the human counterparts of these genes interact with the RNA Polymerase II-Associated PAF1C factors and mutations in the Arabidopsis PAF1C-associated genes also produce early flowering, thereby linking two important developmental transitions, flowering time and seed dormancy [149]. The *PIL5* promoter is also a target for histone modification, since the EFS methyltransferase increases the level of H3K36me2 and H3K36me3 to promote recruitment of RNA polymerase II and thus enhance *PIL5* transcription [151]. Acetylation is another chromatin modification associated with active gene expression. Mechanisms involving deacetylation of genes with a negative effect on dormancy have been observed. In particular, members of the SNL deacetylation complex regulate key genes involved in ABA, ethylene and auxin pathways. The expression of SNL1 and SNL2 increases gradually during embryo development and seed maturation, causing a decrease in the acetylation level (H3K9/K18 and H3K14) of ABA hydrolytic genes (*CYP707A1* and *CYP707A2*) and some ethylene-related genes (*ACO1* and *ACO4*). This favors higher ABA levels and blocks the ethylene pathway [152]. During imbibition of AR seeds, the expression of SNL1 and SNL2 declines, causing an increase in the acetylation levels of auxin pathway genes (e.g., the auxin importer *AUX1*). Subsequent activation of *AUX1* transcription leads to increased auxin levels and signaling, followed by enhanced cell division that promotes seed germination [153]. The plant homeodomain (PHD) motif-containing EBS is involved in the control of flowering time by binding to H3K4me2/3 and recruiting histone deacetylases (HDACs) to H3 [154]. It was shown that the *ebs* mutant also showed a reduction in seed dormancy independent of DOG1 [155]. In addition, PIL5 recruits HDA15 deacetylase to decrease the H3 acetylation levels of its target gene promoters to repress germination in the dark [156]. Furthermore, the LUH protein, a member of the Groucho family of transcriptional corepressors (known to recruit either HDAC or mediator complexes [157]), acts as PIL5 corepressor in the dark [158]. Another deacetylase, HDA9, negatively influences germination and promotes dormancy. HDA9 is involved in the transcriptional repression of genes related to the transition from seed to seedling during seed development, probably through H3K9 deacetylation [159]. 

The HDA9 function is opposite to that of its homologous genes *HDA6* and *HDA19*, which have been reported to repress embryonic properties upon seed imbibition, probably via H3K9 deacetylation [160]. This is another indication that active chromatin modifications are required to promote germination. A similar effect is produced by Arabidopsis PHD-domain H3K4me3-binding AL proteins. AL6 and AL7 are able to interact and build complexes with PRC1 polycomb proteins around H3K4me3 marks, leading to a switch from the H3K4me3-associated active to the H3K27me3-associated repressive transcription state of genes associated to seed development (e.g., *ABI3*, *DOG1*, *CRU3*, *CHO1*) during seed germination [161]. Additional repressors of seed maturation genes have been found in vegetative organs and germinating seeds [162]. These include the polycomb EMF2-PRC2 complex combined with the SDG8 methyltransferase, which are required to maintain the H3K27me3 repressive mark in seedlings [163]; two ZRF proteins that contribute to PCR1-mediated repression by binding to monoubiquitinated H2As and H3K27me3 [164]; the SUVH5 methyltransferase mediating repressive dimethylation of H3K9 [165]; LDL1/2 demethylases, which potentially remove activating histone modifications (H3K4me2/3) from seed dormancy genes [166,167]. Finally, deacetylation of H2B by the HD2B deacetylase is associated with reduced dormancy and increased GA levels in imbibed seeds. *HD2B* expression is upregulated by cold or AR in accessions of Arabidopsis with low dormancy (Columbia-0; Col-0), and correlates with a reduction in the expression levels of GA inactivating genes (*GA2ox2*) as well as increased expression of GA biosynthetic genes (*GA3ox1/2*). This upregulation of *HD2B* expression is significantly suppressed in Arabidopsis accessions showing high dormancy (Cape Verde Islands; Cvi-0) [168]. Several TFs have also been found to recruit some of these modifiers to negatively regulate specific dormancy-associated chromatin locations. The BES1 TF is able to form a transcriptional repressor complex with the TPL corepressor and HDA19 at the *ABI3* locus [169]; the SCL15 TF recruits HDA19 at a subset of embryonic-specific loci in vegetative tissues [170]; the HSI2 TF recruits HDA6 to repress seed maturation genes upon post-germination [171]. Some of these chromatin changes may also affect TF loci. The repressive role of the H3K27me3 mark on specific negative regulators of germination such as DOG1, DAG1 or SOM, seems to be particularly important for developmental phase transitions, especially from the embryonic to seedling stage [172,173]. During germination, the SANT domain-containing protein PWR, previously reported to act in a complex with HDA9 in leaves [174], suppresses ABI3-dependent *SOM* transcription by accelerating histone H3 deacetylation levels and H2A.Z deposition at the *SOM* locus. Seed imbibition under high temperature stress blocks *PWR* transcription and triggers secondary dormancy [175]. H3 deacetylation of *SOM* is also a target in carbon monoxide (CO) signaling. Light and PhyB-mediated germination increase transcription of *HY1* oxygenase for CO production (a molecular signal with a positive role in stress-mediated germination) by inducing antioxidant metabolism as well as the degradation of storage reserves [176,177,178]. CO signaling recruits HDA6 to the promoter of *SOM* to decrease its expression by H3 deacetylation [178].

Derepression of gene expression is also required during germination. Two histone arginine demethylases (JMJ20/JMJ22) have been shown to be positive regulators of light-induced germination through the removal of repressive H4R3me2s at *GA3ox1/GA3ox2* resulting in increased GA levels. This regulation is mediated by light, as *JMJ20/JMJ22* are directly repressed by SOM when PhyB is inactive [179]. 

Chromatin structure can also be changed by ATP-dependent remodeling complexes [180]. Several of their components have been found to repress dormancy genes or embryonic traits in post-germinative growth, such as BRM (SWI2/SNF2 subgroup ATPase [181]) and PKL (CHD3 class [182]). In seeds, BRM promotes germination and directly associates with the promoters of two positive regulators of GA signaling, *GA3ox1* and *SCL3* TF [183]. PKL is required for about 80% of the gene expression changes triggered by GAs [184]. On the other hand, the repression of the *CHR12/23* genes (SWI2/SNF2 subgroup ATPases) is required for full germination since their overexpression represses germination by elevating the levels of maturation-related genes [185]. 

DNA methylation is another epigenetic modification usually associated with transcriptional repression. Extensive gain of CHH methylation during seed development and drastic loss of CHH methylation during germination have been observed. These findings hint at dynamic DNA methylation reprogramming events as probable mechanisms regulating both developmental stages [186]. Such notion was corroborated in another study detecting large-scale CHH demethylation levels towards the end of germination. However, it cannot be ruled out that these events are the result of passive demethylation, as they coincide with the onset of DNA replication and hence could not be strongly associated with gene expression changes [187].

### 2.5. Germination Control by Small RNAs and Post-Transcriptional Regulation

Small RNAs are known to regulate gene expression in developing and germinating seeds. Several gene mutations related to small RNA biogenesis display severe defects in embryogenesis and seed development. Likewise, mutants of miRNA coding genes have altered levels of regulatory genes controlling seed dormancy and germination [188,189]. Transcriptional profiling of miRNAs during seed production of two Arabidopsis accessions with contrasting dormancy levels has revealed that the more dormant accession contains higher levels of miRNAs. Although computational analyses identified specific TFs involved in hormone signaling as putative miRNA targets, these predictions remain to be validated [190]. Additional studies on the role of miRNAs upon seed imbibition have found several miRNAs belonging to different families which are up- and down-regulated during this process [191,192,193]. Various miRNAs have also been described to influence seed germination under various abiotic stresses [189] and specific links have been proposed between miRNAs and TFs mediating hormone signaling during seed germination [194,195,196]. In addition, miR166 has been shown to contribute to the repression of maturation and dormancy genes in vegetative tissues [197]. On the other hand, many maternally expressed siRNAs transcribed by the NRPD1 polymerase (the largest subunit of RNA polymerase IV) during seed development have recently been found to regulate temporal and spatial expression of endosperm genes [198,199,200,201]. For instance, the regulation of *AGL40/91* TF mRNA levels by several siRNAs is responsible for changes in seed size [201]. One type of siRNAs, the trans-acting siRNAs (ta-siRNA), have also been implicated in plant development. Ta-siRNAs are generated from the Trans-Acting SiRNA locus (TAS) gene resulting in non-coding transcripts through specific miRNA guided cleavage [189,202]. miR390 is required for the processing of a functional ta-siRNA-ARF that targets and negatively regulates auxin response TFs (ARF2, ARF3, and ARF4) in early stages of seed germination, indicating a crosstalk of ta-siRNAs and miRNAs in this process [193]. 

A less studied molecular event controlling germination is mRNA stability. It is well known that dry seeds accumulate extant RNAs whose abundance change during AR towards a “germination-friendly” transcriptome [203,204,205,206]. Taking into account that very low or no transcription is supported by quiescent seeds, it makes sense that the up and down-regulation observed for sets of AR regulated genes correlates with their mRNA decay rates [207,208,209]. Moreover, several groups have obtained evidence supporting a role of active mRNA degradation in the control of dormancy/germination responses. Thus, a 3’-5’ exonuclease (RRP41L), a subunit of the core exosome in Arabidopsis, is responsible for cytoplasmic degradation of specific mRNAs related to ABA signaling. Therefore, *rrp41L* loss-of-function seeds and seedlings showed ABA-hypersensitive phenotypes [210]. Evidence suggesting the involvement of 5’-3’ RNA decay in the control of dormancy and germination has also been found. It was observed that two 5’-3’ RNA decay mutants (*xrn4* and *vcs8*) have altered and opposite dormancy and germination phenotypes. Moreover, transcript abundance of specific ABA/GA metabolism and signaling genes was modified accordingly to their phenotypes, suggesting that they could be direct targets of those exoribonucleases [208]. In another study, several loss-of-function mutants of the Arabidopsis 5′-3′ mRNA decay machinery were found to have enhanced ABA sensitivity. While the DCP5 (decapping) component of this machinery affected seed germination, the LSM1 (decapping activation) and XRN4 (exonucleolytic degradation) components impacted early stages of vegetative growth. The DCP5 and LSM1 components were found to have a negative effect on mRNA and protein levels of specific PYL/PYR ABA receptors, but only the *lsm1* mutants showed higher levels of the SnRK2.6 kinase mRNA and protein [211]. It is worth mentioning that not all the components of the 5′-3′ mRNA decay machinery showed the same responses at the phenotypic and molecular level, and that discrepancies were observed for the *xrn4* mutant in two studies [208,211]. Therefore, the degree of functional/genetic redundancy under different growth conditions thus remains an open question. Thus, the existence of additional mechanisms controlling mRNA turnover in seeds, like the targeted oxidation reported in sunflower [212], cannot be ruled out. 

Several studies have found that differential recruitment of mRNAs by ribosomes adds an extra layer of regulation in both dormant and nondormant seeds [213,214]. More dynamic changes in polysomal occupancy were observed for nondormant seeds upon imbibition than for dormant seeds. GC content and the number of upstream open reading frames (uORFs) were identified as transcript features with a possible role in this selective translation. Polysomal RNAs associated with germination fell essentially into the cell wall, hormone metabolism, and redox pathway categories, while mRNAs related to stress responses and hormone metabolism pathways were associated with polysomal RNAs connected to dormancy. These results along with the absence of correlations between transcriptome and translatome led to propose that the transition from dormancy to germination is regulated mainly at the translational level [214]. In agreement with these results, large changes in polysome occupancy were found to occur upon seed imbibition, being mainly associated to the transition from dry to hydrated seed (6 h after imbibition; hai) and from 26 hai to a germinated seed (48 hai) [215]. However, the same authors found that polysomal mRNAs do not change between imbibed dormant and AR seeds treated with a transcriptional inhibitor. Since both conditions block germination, this result suggests a relevant role of transcriptional regulation and transcript abundance in controlling the germination onset [216]. These apparently contradictory results underscore that more information is needed to assess the specific roles of transcription and translation in the control of germination. In dry seeds, ribosomes are mainly present in the monosome form and certain transcript features (i.e., uORFs, length, stability) differed significantly between the polysome populations associated to specific germination stages [215]. It has been shown that most mRNAs in dry seeds are stored as monosomes forming complexes with mRNA-binding proteins, stress granules (SG), and P-body proteins. About 17% of those mRNAs are translationally up-regulated during seed germination and transcribed during seed maturation [217]. Moreover, mRNAs are not likely to be translated when found as monosomes, or are translated at low levels, since levels of translation usually correlate with ribosome density [218]. All these pieces of evidence suggest a possible scenario in which environmental conditions may modulate regulated packing of mRNAs in dry seeds to impact on their translational fate and rate upon imbibition.

Lastly, regarding post-translational modifications, changes in the phosphorylation levels of proteins during seed germination have been reported in rice, mainly during the first 12 hai. The first 12 hai are critical not only for posttranslational processes but also for transcription and metabolic changes, because the decision making for germination occurs during this period in rice [219]. Moreover, alterations in the phosphorylation/dephosphorylation patterns affect germination [219,220,221]. Besides the changes in phosphorylation status of early components of the ABA signaling pathway previously described, additional phosphorylation events have an impact on germination. FyPP1 and FyPP3 PP6 phosphatases act antagonistically with SnRK2 kinases, dephosphorylating and destabilizing ABI5 [222]. The Raf10 and Raf11 MAP3Ks are positive regulators of dormancy and *ABI3* and *ABI5* expression [223]. Specifically, Raf10 phosphorylates subclass III SnRK2s, which in turn phosphorylate ABI5, ABF2 and ABI3 TFs to enhance their activity [224]. TAP46 is a PP2A phosphatase-associated protein that binds and stabilizes the active phosphorylated form of ABI5, preventing its PP2A-mediated dephosphorylation [225]. Phosphorylation also affects GA signaling. Under salt stress, the GARU E3 ubiquitin ligase suppresses germination by ubiquitination of the GID1 GA receptor. The GID1-GARU interaction is counteracted by the phosphorylation of GARU by the TAGK2 Tyr-kinase [226]. DELLA stability is also associated with phosphorylation, since TOPP4 PP1 phosphatase directly binds and dephosphorylates the RGA and GAI DELLA proteins, promoting their GA-dependent destabilization [227]. The MYB44 TF activity on germination is also dependent of its phosphorylation by MPK3 and MPK6 kinases [228], conversely to RAV1 TF, which is deactivated upon phosphorylation by SNRK2 kinases [89]. Protein ubiquitination is another important protein modification known to negatively affect the stability of proteins with a crucial role in germination, such as DELLAs [14,229], ABI3 [230] and ABI5 [231,232]. Changes in ubiquitination were detected in more than 1000 proteins during rice seed germination and most changes occurred at 12 hai, as observed previously for phosphorylation [233]. Sumoylation has also been demonstrated to play a role in this process by providing stability to ABI5 and MYB30 TFs. Sumoylation protects ABI5 from degradation but makes it inactive [234], suggesting a protective role by maintaining a degradation-resistant inactive pool of ABI5 in the absence of ABA [235]. MYB30 is a negative regulator of ABA responses that seems to provide a balance for the positive regulation exerted by ABI5. Interestingly, both regulators are sumoylated at specific amino acid residues by the same SUMO E3 ligase (SIZ1) [234,236]. ABI5 sumoylation site is in the same domain (K391) as the lysine residue required for KEG-E3-ligase-dependent turnover (K344) [235]. This suggests that ABI5 sumoylation or ubiquitination depends on a direct physical competition of enzymatic activities. Other posttranslational modifications in key regulators of plant growth and development may be important in germination. It is the case of DELLA O-fucosylation and O-GlucosylNAcetylation. RGA DELLA protein interactions with BZR1, PIF3 and PIF4 TFs is promoted by mono-O-fucosylation mediated by the O-fucosyltransferase SPY [237]. On the contrary, RGA interactions with BZR1, PIF3, PIF4 and JAZ1 TFs are inhibited by O-GlucosylNAcetylation mediated by the SEC O-GlcNAc transferase [238]. 

## 3. Genetic Control from Dormancy to Germination Stages

### 3.1. Dormancy

The interaction between the maternal environment and the genetic makeup of the mother plant will determine primary seed dormancy levels during seed maturation. One of the regulatory routes for the establishment and maintenance of physiological dormancy involves the ABA/GA hormonal balance [10,239,240]. Most mutations altering the metabolism, perception and early signaling of these hormones show effects on dormancy levels [239,241,242]. Additional regulators of this hormonal crosstalk have been described. It is the case of ABI4 TF, which increases ABA/GA balance in freshly-harvested seeds and post-germinative stages through direct binding to promoters of some of their metabolic genes [243,244]. The *CHO1* TF gene also contributes to ABI4-mediated regulation [245,246]. MYB96 TF also increases ABA/GA balance and seed dormancy by directly activating *NCED2* and *NCED6* and indirectly repressing *GA3ox1* and *GA20ox1* expression [247]. DOF6 TF promotes dormancy by enhancing ABA-related gene expression [248] and by activating *GATA12* TF expression upon complex formation with the RGL2 protein [249]. Other proteins regulating dormancy are involved in feedback hormone control. The *AtSdr4L* gene encodes a protein of unknown function which is required for the negative feedback control of GA biosynthetic genes, with an impact on dormancy and germination [250]. The WRKY41 TF directly upregulates *ABI3* expression, a function requiring ABA but subjected to negative feedback regulation when the concentration of hormone is sufficiently high [251]. Additionally, circadian clock genes have been shown to play roles in dormancy control [252]. For instance, RVE1 and RVE2 TFs accumulate during seed development to promote seed dormancy but their transcription is repressed by light-mediated activation of PhyB to allow germination [253]. This regulation is counteracted by the ability of RVE1 to interact with RGL2, reducing its interaction with the SLY1 F-box protein and increasing RGL2 stability. In return, RGL2 enhances RVE1 transcriptional activity, which directly represses *GA3ox2* expression [253,254]. 

Besides the hormonal control of dormancy, other pathways have been linked to this process, like those involving *DOG1* and *RDO5*. These dormancy-specific genes were identified by quantitative trait loci analyses (QTL) of natural variation in Arabidopsis [255,256,257]. DOG1 was identified as a key effector of dormancy and has therefore been intensely characterized in recent years [258,259,260]. Its expression in seeds is controlled at four different levels, namely, transcriptional elongation [150], alternative splicing [261,262], alternative polyadenylation [263], and transcriptional suppression by a non-coding *cis* antisense transcript [264]. Different genetic and transcriptomic analyses suggested that *DOG1* exerts its dormancy function in parallel to, but independent of ABA function. Among other pieces of evidence, it has been observed that the non-dormant mutant *dog1* has a normal germination sensitivity to treatments with exogenous ABA [256] and that a high accumulation of ABA or DOG1 protein in the seed cannot compensate for the absence of function of DOG1 or ABA metabolism genes, respectively [265]. These results indicate that both routes are required for an efficient block of dormancy release [265]. Besides dormancy, a genetic interaction between DOG1 and ABI3 has been described during seed maturation, as well as the control of *ABI5* expression by DOG1, revealing a dual role for DOG1 in dormancy and seed development [266]. 

Lately, new convergences have emerged between ABA and DOG1 routes regarding dormancy [260]. DOG1 binds to AHG1 and AHG3, two clade A protein phosphatases 2C (PP2Cs) [267,268] which negatively regulate of ABA signaling and dormancy [269,270,271]. Both phosphatases have redundant roles in dormancy release and are epistatic to *DOG1* [267,268]. It seems that DOG1 has a role in increasing ABA sensitivity through an AHG1 and AHG3 sequestration mechanism, analogous to the perception and initiation of ABA signaling [260,267]. Unlike AHG3 and other members of PP2C, AHG1 is resistant to inhibition by PYR/PYL/RCAR receptors [272]. Some authors have argued that by sequestering this phosphatase, DOG1 might play a role in safeguarding ABA signaling to ensure dormancy until it is inactivated after AR. This would explain why overexpressing DOG1 in ABA-deficient mutants or increasing ABA levels in the *dog1* mutant provoke reduced dormancy [265]. Likewise, the activity of non-sequestered PP2Cs in these ABA-deficient mutants would be sufficient to promote germination [260,267]. It has been recently discovered that DOG1 is a heme-binding protein and such binding is essential for its functionality in dormancy [268]. This may establish a role for DOG1 as an integrator of environmental signals, since heme-binding proteins act as oxygen and NO sensors [260,268].

In addition to the roles in dormancy and seed maturation, other functions have been identified for DOG1. One of them relies on the regulation of DOG1 expression by temperature with an impact on flowering time and dormancy release. Thus, DOG1 is able to modify the levels of two antagonistic miRNAs: miR156, which delays flowering and dormancy release, and miR172, which produces the opposite effect [273]. This coordinated regulation of two developmental phase transitions seems to conform a plant strategy to adapt its life cycle to seasonal environmental conditions [265,273,274,275,276,277]. Finally, in addition to its functionality in reproductive and germinative growth, a role has been proposed for DOG1 in vegetative growth based on the drought-sensitive phenotypes observed in its loss-of-function mutants [278]. Dormancy and drought responses show many similarities at the molecular level regarding ABA signaling. The antisense of DOG1, *asDOG1/1GOD*, silences *DOG1* expression in seeds and leaves, causing dormancy release [264] and drought responses [278], respectively. Upon ABA accumulation, *DOG1* transcript levels increase due to suppression of *asDOG1* expression [274,278,279]. 

RDO5/DOG18/IBO is, together with DOG1, another dormancy-specific gene identified by QTL analyses [257,280,281]. As *dog1*, the *rdo5* mutant shows loss of dormancy, identifying RDO5 as a positive regulator of this process [280]. RDO5 encodes a PP2C without phosphatase activity which probably controls phosphorylation levels by hampering dephosphorylation during imbibition [281,282]. In addition, RDO5 seems to act independently of ABA, since its loss of function does not affect ABA levels or sensitivity to the hormone [280], and does not produce changes in phosphorylation levels of ABA signaling regulators [282]. 

In addition to the main regulatory routes (ABA, DOG1, RDO5), other hormones and their associated molecular mechanisms have been involved in dormancy control. Ethylene reduces dormancy and improves seed germination in several plant species by counteracting ABA effects through the regulation of ABA metabolism and signaling pathways [283,284,285]. Recently, one of the mutants previously identified for exhibiting a reduced dormancy phenotype, *rdo3* [286], was shown to be a loss of function of the *ETR1* ethylene receptor [287]. Although ETR1 does not require the canonical ethylene signaling pathway to act in this process, it is involved in the induction of ABA signaling genes [288]. Thus, when ETR1 function is lost, *ERF12* TF is upregulated by an unknown transduction pathway which probably involves MAP kinases, and forms a repression complex with TPL that binds the *DOG1* promoter and represses its expression [287]. 

Auxins also have a role in dormancy in an ABA-dependent manner, since treatment with exogenous indole-3-acetic acid (IAA) in combination with ABA enhances dormancy. In addition, *ARF* TF mutants, components of the auxin response, have reduced dormancy levels and are less sensitive to ABA treatments [289]. ARF10 and ARF16 have been identified as positive regulators of dormancy through indirect regulation of *ABI3* expression [289]. These results seem contradictory with those obtained by another study, which describes that in dormant seeds of near-isogenic Arabidopsis lines carrying the Cvi-0 *DOG1* loci introgressed in a *Landsberg erecta* (Ler) genetic background, the latter being a less dormant accession than Cvi-0, tryptophan-dependent auxin biosynthesis and related pathways are strongly repressed compared to germinating seeds [216].

One interesting aspect of dormancy is the fact that forest fires generate chemical signals that can stimulate the germination of certain dormant seeds in the soil. These compounds include cyanohydrins [290] and karrikins [291,292,293]. Arabidopsis and other seeds of *Brassicaceae* respond to karrikins [294] by using the KAI2 receptor, a paralogue of the D14 strigolactone receptor [295]. The binding of karrikins to KAI2 leads to their interaction with the MAX2 F-box protein, which targets TFs such as SMAX1 for degradation [296]. Consequently, the loss-of-function mutants *kai2* and *max2* show enhanced dormancy [295,297,298,299], probably by negatively affecting *CYP707A* expression [300]. Such phenotype is reverted by the loss of *SMAX1* function [296]. Interestingly, while GAs or nitrate always stimulate dormancy release, karrikins promote dormancy when combined with abiotic stresses such as NaCl, mannitol and elevated temperature [298]. This dual role of karrikins, as dormancy enhancers or repressors depending on environmental conditions, suggests that karrikin signaling factors may function as safeguards to prevent germination until conditions are optimal.

Effects on dormancy by jasmonate (JA) had rarely been reported. The best studied case is that of the JA precursor 12-oxo-phytodienoic acid (OPDA), which produces an increase in dormancy upon accumulation in the *comatose* (*cts*) mutant through a positive feedback with other positive dormancy regulators (i.e., ABA, RGL2 and MFT) [301,302]. Additionally, the maternal herbivory, defined as the maternal experience of herbivore feeding during flowering and seed development seems to have an effect on dormancy through JA pathway regulation [303]. The accumulation of JA-isoleucin (JA-Ile) during seed development, either by maternal herbivory or overexpression of the AOS JA biosynthesis enzyme, produces a reduction in dormancy. This phenotype is associated with increased GA content and reduced ABA sensitivity, a response absent in a JA-Ile-deficient (*jar1-1*) mutant [303].

Herbivory is not the only process that regulates dormancy maternally. The level of seed dormancy is highly influenced too by the environmental conditions experienced by the mother plant. In this case, FLC TF [304,305,306], RGL2 and the phosphatidylethanolamine-binding protein FT [305,307] play important roles, unlike other key dormancy proteins such as DOG1 [305]. In siliques, the expression of *FT* and *FLC* responds to temperature in the maternal tissues but not in those of the seed. Accordingly, under cold conditions, the maternal FT protein expressed in the silique phloem controls dormancy through inhibition of proanthocyanidin synthesis in the seed, thus altering the levels of testa tannins [305,308]. Maternal inheritance is also evidenced during genomic imprinting, the preferential expression of a given parental allele over the other [309]. The importance of this process in the maternal inheritance of seed dormancy has recently been described. A set of genes were found to be imprinted in endosperm cells and the maternal alleles were preferentially expressed upon seed imbibition [310]. For instance, in the case of the *ALN* gene, cold stimulates the differential methylation of the promoter of the paternal allele to promote dormancy [311].

### 3.2. After-ripening and Longevity

In dry seeds, the cytoplasm reaches a highly viscous glassy state which severely limits molecular diffusion and the occurrence of chemical and enzymatic reactions, but maximizes seed survival [312,313]. Despite apparent inactivity, seeds continue to undergo physiological changes such as loss of dormancy, or even loss of longevity, defined as the total time span during which seeds remain viable.

Primary dormancy is acquired during seed maturation and is gradually lost during dry storage (or AR), a process that depends on the relationship between seed moisture content and temperature [212,314,315,316,317]. Loss of dormancy has been associated with an accumulation of reactive oxygen species (ROS), which would lead to oxidation of proteins and mRNAs [212,318,319,320]. Non-enzymatic oxidative reactions have been associated with low moisture content (below ~0.12 g H_2_O g dw^–1^) while metabolic reactions would prevail under high moisture conditions [314]. No active transcription is required in Arabidopsis during the AR process [321]. Like in wheat and sunflower, there is hardly any change in the abundance of transcripts between dry dormant and AR non-dormant seeds [204,322,323]. In addition, although a clear correlation between transcriptome and translatome upon seed imbibition has not been found, there is a selective recruitment of mRNAs into polysomes [213,214]. Thus, in Arabidopsis, one-third of the polysome-associated transcripts are similar at 16 and 24 hai in dormant seeds, while only around 4% are common in nondormant seeds between 16 and 24 hai [214]. It has also been suggested that oxidation of specific mRNAs during AR might reduce their translation during seed imbibition, even if they are still present in the transcriptome [212,322,323,324]. In the same line, specific recruitment of mRNAs is thought to be based on features of the 5’-UTR [214,325]. These findings, along with the fact that the germination program is activated in non-dormant seeds after 8–24 hai, suggest that there is a developmental checkpoint during the first hours of imbibition [325]. In this way, a selective translation of mRNA during imbibition will maintain or not the inhibition of germination, depending on the oxidative imprinting of the seed. Certain components may be particularly sensitive to oxidation as part of such imprinting.

When environmental conditions impose a long block on germination, the viability of dry seeds, and therefore their longevity, can be reduced due to excessive oxidation-derived damage of molecular components. Seeds have different mechanisms to favor longevity and some genetic factors involved in this process have been identified by QTL analysis using natural variation in Arabidopsis [326,327,328]. Many of these factors have functions related to protection of different biomolecules from ROS [329,330,331,332]. Among these factors are vitamin E (tocopherols and tocotrienols), which prevents the non-enzymatic oxidation of lipids [333], protein L-isoaspartyl methyltransferase, with a role in the repair of damaged proteins [334], metallothioneins [335], methionine sulfoxide reductase [336], lipoxygenase [337], the glycosylase/apurinic/apyrimidinic lyase DNA [338], and the prolyl isomerases rotamase FKBP 1 and 2 [339]. Noteworthy are the mitochondrial NADH dehydrogenase ferric-chelate reductase 1 [326] and the NADP-malic enzyme 1, whose function losses produce a reduction in longevity, and, for the latter mutant, enhanced protein carbonylation in aged seeds [340,341]. Likewise, it has been observed that seed-storage proteins buffer seed biomolecules from oxidative stress [328]. One of the cellular components damaged during prolonged dry state is DNA, with double-strand breaks (DSBs) being rate-limiting for germination [342]. Plants have a specific response to integrate the germination progress of aged seeds with the monitoring of genome integrity. This mechanism is the DNA damage signaling or DNA damage response, in which the checkpoint kinases ATM and ATR play key roles [343,344]. Aged *atr* and *atm* mutant seeds germinate faster than aged WT seeds and show earlier activation of DNA replication and extensive chromosomal abnormalities. Thus, ATM and ATR contribute to the control of germination by inhibition of DNA replication in aged seeds upon imbibition, partly through the transcriptional up-regulation of the SMR5 cell cycle inhibitor by ATM [343]. 

Other regulatory proteins with an impact on these processes have also been identified. This is the case for PhyA and PhyB phytochromes [345], the RSL1 E3 ubiquitin ligase [346], and the TFs CDF4/DOF2.3 [345], ATHB25 [347] and COG1/DOF1.5 [345]. The composition and structure of the seed coat are critical factors for seed longevity by providing chemical and mechanical protection [348]. The longevity effects of mutations in ATHB25, as well as in phytochromes and COG1, correlate with accumulation of mucilage and suberine, respectively [345,347]. The loss of *TT10* laccase function, which is involved in seed coat lignin biosynthesis [349,350], also produces a reduction in longevity [351]. Additionally, because longevity is induced during seed maturation [352,353,354], mutations altering seed development (e.g., *lec1*, *lec2*, *fus3*, *abi3* [326]), and *DOG1* (e.g., *dog1* [256,266]), also produce longevity defects.

### 3.3. Seed Bank and Secondary Dormancy

Most studies on germination are made under controlled laboratory conditions with minimal environmental variation. However, seeds shed in the field are exposed to a journey of uncertain duration under shifting environmental conditions. During the year, seeds in the soil (seed bank) are repeatedly imbibed and dried, suffering temperature shifts during variable periods of time. Under this state of continuous change, the seed bank must be capable of sensing external conditions and adjusting their germination potential accordingly. That is why non-dormant seeds can retrieve the dormancy program as a protective measure upon encountering inadequate conditions. This ability is called secondary dormancy and can be lost and gained repeatedly until germination occurs or viability is lost. The molecular processes underlying this dormancy cycling in the seed bank have been less studied than those involved in primary or secondary dormancy in the laboratory. However, several publications have started shedding light on this ecological process from a physiological [355] and a molecular standpoint [316,356,357]. For a winter annual plant, the beginning of winter coincides with an increase in dormancy. This dormancy correlates with higher ABA/GA ratios supported by enhanced expression of ABA biosynthetic and GA catabolic genes, followed by a subsequent increase of gene expression related to ABA signaling [358,359]. This leads to a stage of deep dormancy, reinforced by the increased expression of *DOG1* and *MFT* [358,359,360]. Reversion of these events is linked with dormancy release, starting in spring and leading to a shallow dormancy state [358,359]. This cycle of transitions between deep and shallow dormancy corresponds to a temporal sensing of seasonal changes. Germination is also fine-tuned during shallow dormancy stages by increasing sensitivity to light and nitrate and upregulating DELLA expression (RGL2/RGA). In this way seeds can couple spatial sensing information with the onset of germination [358,359,360].

### 3.4. Regulation of Germination from a Spatial and Mechanical Perspective

Another important aspect of seed germination relates to the interplay of mechanical forces between seed tissues. The ability of seeds to germinate is thought to result from a balance between physical restrictions imposed by the embryo-surrounding tissues (testa and endosperm) and the ability of the embryo to grow and protrude [3,361]. The decline in the mechanical resistance of the micropylar endosperm, which covers the radicle tip, leads to endosperm weakening and appears to be a general prerequisite for radicle protrusion (germination *sensu stricto*) [362,363,364]. Previous studies with non-dormant seeds have shown that the expression of many Cell Wall Remodeling Enzymes (CWREs) are upregulated by GAs, in correlation with endosperm weakening and embryo radicle protrusion. These findings point at the composition of cell walls and their mechanical properties as relevant targets to control germination [3,363,364,365].

Xyloglucans (XyGs), the major components of hemicelluloses in the primary cell walls, have been found to play a role in wall remodeling. Mutant seeds lacking functional XYL1, an α-xylosidase involved in XyG biosynthesis, were able to germinate on PAC and had reduced dormancy, thermoinhibition-resistant germination and alterations in specific genes involved in ABA/GA metabolism, all characteristics resembling ABA-deficient mutants [366,367]. In addition, the mutants showed changes in the composition of endosperm cell walls resulting in reduced strength, which supports the notion that *XYL1* is a negative regulator of germination [366]. Moreover, different results have shown a localization of XyGs compatible with a role in germination. Thus, immunolocalization experiments in germinating seeds indicated a reduction of xyloglucans (XyG) in the elongation zone of the embryonic axis but not in the cotyledons or root tips [366]. A promoter:GUS fusion also revealed low *XYL1* expression in endosperm, as expected for a tissue required to reduce mechanical resistance to embryo growth upon imbibition [367]. XTH endotransglycosylases/hydrolases are another type of XyG-related enzymes that can cleave and reconnect XyG chains and several of them are upregulated upon seed imbibition of non-dormant seeds [3,365]. One of them, *XTH31*, is thought to reinforce endosperm cell walls, since its loss of function led to faster germination [365]. 

Other important components of cell walls, pectins and pectin methylesterases (PMEs), have been associated with promoting seed germination mainly acting on testa permeability [368]. Although PME activity is usually linked to enhanced pectin de-esterification and increased wall rigidity, it can produce the opposite effect in combination with appropriate enzymatic activities [369]. Genetic redundancy may be a problem when ascribing roles to PME members in the regulation of germination. One example is the *pme58* mutant that shows normal germination despite having altered seed-coat mucilage structure [369].

Wherever cells are growing or modifying their walls, one or more expansin genes are usually active, promoting primary cell wall relaxation [370]. These proteins are thought to specifically modify the interactions between XyG and cellulose microfibrils. *EXPA2* encodes a GA-induced endosperm-specific α-expansin with a proven genetic role in enhancing germination [371].

In addition to cell walls, a thick cuticle layer was discovered to be tightly associated with the outer surface of the endosperm cell layer of Arabidopsis seeds [372]. It was later found that this cuticle had a maternal origin, deriving from a specific layer of the ovule integuments that becomes associated to the endosperm at late stages of seed development [373]. This cuticle regulates permeability and has an impact on seed dormancy and viability. Mutants defective in cutin biosynthesis (e.g., *lacs2*, *bdg1*) are unable to block endosperm cell expansion and testa rupture under adverse conditions [372]. It has been suggested that this cuticle could prevent integument-endosperm fusion during seed development, keeping unwanted developmental signals from entering the endosperm [373]. Indeed, it has been shown that the endosperm plays an important role in linking the perception of environmental signals to the control of dormancy and its cycling by modulating ABA released to the embryo and controlling gene expression [63,64,357]. 

**Figure 2:**
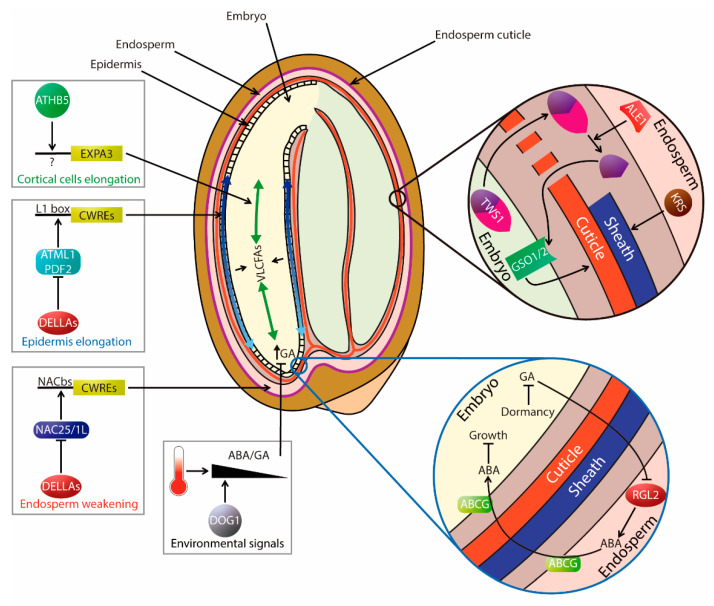
Spatial and mechanical regulation of seed germination. The seed coat protects living tissues from mechanical and oxidative damage. In addition, a cuticle layer associated with the outer surface of the endosperm regulates permeability, modulating seed physiology. Despite these seed coverings, embryo inner cells are able to continuously sense the environment and decide when to germinate. A specific area within the embryonic radicle acts as a decision-making center inducing changes in ABA/GA in response to variable temperature. In the endosperm, *DOG1* couples temperature with the regulation of GA metabolism to control *CWRE* gene expression required for the weakening of cell walls. Endosperm also controls embryo growth in dormant seeds by RGL2-dependent release of ABA and seed specific ABCG transporters. Once germination is triggered, there is an interplay of mechanical forces as the embryo pushes against its surrounding tissues. GA biosynthetic and expansion-promoting gene expression is induced very early in the radicle tip upon imbibition. Due to mechanical constraints, cell expansion is observed mainly in the upper limits of the radicle, extending afterward along the embryonic axis. This expansion is required for germination and depends on GA-responsive epidermis-specific gene expression mediated by two HD-ZIP proteins, ATML1 and PDF2. They activate *CWRE* and *VLCFA* genes to coordinate epidermal cell expansion with that of inner tissues. ATHB5 also controls cell expansion, but mainly in cortical cell layers of the upper embryonic axis. Cell expansion along the embryonic axis contributes to testa rupture and germination. Endosperm cells elongate at different rates to accommodate embryo growth. This process is controlled mainly by GA signaling mediated by NAC25 and NAC1L, which upon the perception of an unknown embryonic signal activates *CWRE* expression. Communication between embryo and endosperm to coordinate germination also occurs during seed development by two mechanisms: (1) A peptide-mediated bidirectional signaling controls the deposition of an embryo cuticle to minimize water loss (embryo secreted TWS1 peptide; endosperm-specific ALE1 subtilase; GSO1/GSO2 receptor-like kinases); (2) An endosperm-derived peptide triggers deposition of the embryo sheath, which facilitates coat shedding and seedling establishment (KRS endosperm-specific peptide).

Several publications have established a link between germination and cell wall remodeling and cell expansion (Figure 2). It was found that GA signaling in the Arabidopsis embryo epidermis along the embryonic axis is required for proper germination. A DNA sequence (L1 box) conserved in the promoters of epidermis-specific genes is bound by two homeodomain (HD-ZIP) TFs (ATML1 and PDF2) and mediates GA-induced transcription of these genes. Since the function of these TFs is blocked by physical interactions with DELLA proteins, increased GA levels produced upon imbibition would cause DELLA degradation. Subsequent release of these TFs will enhance cell elongation and germination mediated by CWRE genes like *EXP8* [59]. The elongation of the epidermal cells is likely to be coordinated with those of inner tissues by activating additional HD-ZIP target genes involved in the biosynthesis of very-long-chain fatty acids (VLCFAs) [59], a mechanism demonstrated for vegetative stages [374]. Indeed, imaging studies on germinating embryos demonstrated that cell surface area increases mainly in the epidermis and moves progressively towards inner layers [60]. Firstly, cell expansion in the lower embryonic axis contributes to testa rupture and then expansion of its upper part promotes protrusion of the radicle through the seed coverings [60]. By combining spatio-temporal gene expression information with promoter analyses, another homeodomain TF (ATHB5) was found to control the expression of the expansin gene *EXPA3*. This control takes place mainly in cortical cell layers of the upper embryonic axis, suggesting a tissue-specificity and partially overlapping roles of homeodomain genes [59,60]. Many morphological features related to elongation events and cell wall remodeling in the embryo seem to be conserved between species [375,376,377]. Moreover, the epidermal HD-ZIP-DELLA-L1 box regulatory module was found to be conserved in cotton where it controls fiber cell elongation [378], indicating that it has been recruited by other developmental stages. Another study demonstrated that expansion-promoting gene expression in embryo radicle tips, including GA biosynthetic genes, is induced very early after seed imbibition (1–3 hai). However, due to mechanical constraints cell expansion is observed mainly in the upper limits of the radicle, which extends along the embryonic axis during subsequent stages of germination. These results are a clear indication that cell geometry and the interplay of mechanical forces between cells have an influence on genetically specified growth [379]. The embryo radicle tip was also found to be an important place where temperature has an impact on gene expression by influencing tissue/cell-specific ABA/GA balance. This dynamic regulation largely determines whether a seed germinates or remains dormant in the soil [61].

Although the structure of cell walls differs between the endosperm and the embryo, cell wall architecture of Brassicaceae and Solanaceae species is similar in the micropylar endosperm [380]. Differential gene expression in the endosperm is concentrated in the micropylar end and involves key genes for cell wall function, many of them induced by GAs [3,58]. Genetic manipulation of specific cell wall components or cell wall-related enzymes in the endosperm is known to have an impact on seed germination [362,365,372,380,381]. DOG1 impacts germination by coupling temperature with GA metabolism to control the CWRE gene activity required for the biomechanical weakening of the endosperm [382]. Endosperm expansion during imbibition was also identified as a necessary key step in the regulation of germination and is controlled by an embryo-initiated gene network [62,382]. By using 3D geometry cell reconstruction, it was observed that all endosperm cells expand during imbibition, but at different rates, to accommodate embryo growth and to facilitate germination. A molecular mechanism underlying endosperm cell expansion was found to be controlled by two NAC TFs (NAC25 and NAC1L) that, when released from repression by the RGL2 DELLA protein, perceived appropriate signals from embryo and activated the expression of a cohort of CWREs [62]. 

Although the regulatory signals that move from embryo to endosperm remain elusive, endosperm-derived signals triggering a deposition of hydrophobic and anti-adhesive barriers on the embryo surface have been recently discovered. Peptide-mediated bidirectional signaling controls the deposition of the embryo cuticle that prevents organ fusion during seed development and excessive water loss in young plants to maximize seedling survival at germination [383,384,385,386,387,388]. Finally, the ZOU/RGE1 TF is also responsible for the production of the KRS endosperm-specific peptide, which triggers deposition of the embryo sheath, a layer of endosperm-derived material rich in extensin-like molecules [389]. This sheath is deposited outside the embryonic cuticle reducing the adhesion to the endosperm during germination and thus facilitating seed-coat shedding and rapid seedling establishment [390].

## 4. Future Directions 

Despite our extensive knowledge of seed biology, many important questions remain to be answered. For example, the contribution of cell division between seed imbibition and seed germination is still unclear. Whereas in Arabidopsis and most species no cell division is observed before germination, genome duplications and activation of cell cycle genes are known to occur at late germination stages, contributing to germination speed [391,392,393,394]. This may be a strategy to guarantee genome integrity by allowing DNA damage responses to scan and repair the genome, but more studies are needed. Another interesting question is how the described transport activities are coordinated and linked to hormone metabolism and signaling. In this respect, the study and development of hormone sensors and tagged hormones [395,396,397] should provide helpful information. Novel transporters may participate in the precise control of hormonal balances in seeds (probably not only for ABA/GA). The fact that mutations in proteins involved in transcriptional elongation only seem to alter seed dormancy and flowering remains a puzzling issue. A link between these two developmental stages has been underscored by mutations in *DOG1* and *FT*, two genes that independently regulate dormancy [273,305]. Despite increasing data on the regulation of germination by mechanisms affecting RNA dynamics, this topic has received much less attention than other molecular layers of regulation. In particular, RNA modifications with a profound impact on RNA activity, such as N^6^ or N^1^ adenosine methylation, have not yet been reported to play a role in seeds. Finally, the discovery of tissue-specific signaling mechanisms and supra-cellular structures is providing a more complex picture of the control of seed germination. Such a picture will be fine-tuned in the near future with studies at the single-cell level.

## Figures and Tables

**Table 1:** Modifiers and remodelers altering the chromatin status of genes promoting dormancy and germination.

**Effect: Promotes Dormancy**			
**Modification**	**Modifier**	**Targets (dormancy genes)**	**Reference**
H2B-Ubq	HUB1/2	*DOG1, ATS2, NCED9, PER1, CYP707A2*	[146]
H3K4me	ATXR7	*FLC*	[149]
H3K36me2/me3	EFS	*PIL5*	[151]
**Modification**	**Modifier**	**Targets (germination genes)**	**Reference**
H3K9/K18/K14deAc	HDA19+SNL1/2	*CYP707A1/2, ACO1/4*	[152]
H3deAc	HDA15+PIL5	PIL5 target genes	[156]
H3K9deAc	HDA9	Photosynthesis genes	[159]
**Effect: Promotes Germination**			
**Modification**	**Modifier**	**Targets (dormancy genes)**	**Reference**
H3K9deAc	HDA6/19	*LEC1/2, FUS3, ABI3*	[160]
H3K4me3 to H3K27me3	AL6/7-PRC1	*ABI3, DOG1, CRU3, CHO1*	[161]
H3K27me3 maintenance	SDG8-EMF2-PRC2	*ABI3, FUS3, LEC1/2*	[163]
H2Aub1 and H3K27me3	ZRF1a/b-PRC1	*ABI3, CRU3, CHO1*	[164]
H3K9me2	SUVH5	*ABA1/3, NCED6, ABI5, DOG* genes	[165]
H3K4me2/3 demethylation	LDL1/2	*DOG1, ABA2, ABI3*	[167]
H2BdeAc	HD2B	*GA3ox1, GA3ox2*	[168]
H3K9deAc	BES1-TPL-HDA19	*ABI3*	[169]
H3K9deAc	SCL15+HDA19	*CRA1, δ-VPE, α-TIP*	[170]
H3K9deAc	HSI2-HDA6-MED13	*LEC1, LEC2, FUS3, ABI3*	[171]
H3deAc and H2A.Z deposition	PWR-HDA9	*SOM*	[175]
H3deAc	HDA6-CO signaling	*SOM*	[178]
**Modification**	**Modifier**	**Targets (germination genes)**	**Reference**
H4R3me2 demethylation	JMJ20/22	*GA3ox1/2*	[179]
ATP-depending remodeling	BRM	*GA3ox1, SCL3*	[181]
ATP-depending remodeling	PICKLE	*GA3ox1, GA20ox1, GID1A, GID1B, SCL3*	[184]

## Supplementary Materials

The following are available online at https://www.mdpi.com/2223-7747/9/6/703/s1, Table S1: Compilation of selected recent reviews on specific aspects of seed biology, Table S2: List of complete gene names in alphabetical order according to their acronyms.
