# Supplementary material for: An Updated Overview on the Regulation of Seed Germination
Source: Plants (Basel). 2020 Jun 1;9(6):703. doi: 10.3390/plants9060703 (PMC7356954; doi:10.3390/plants9060703)
Supplement: Supplementary file 1 [file plants-09-00703-s001.pdf]

An Updated Overview on the Regulation of Seed Germination

Table S1. Selected recent reviews on seed germination-related aspects.

Subject (Keywords)	References
**Generalists**	
ABA, Arabidopsis, dormancy, germination, GA, seed maturation.	[3]
**GAs and ABA**	
GAs, ABA, hormone signaling, seed germination, abiotic stresses, crosstalk of hormone signaling.	[10]
DELLAs, GAs, hormone crosstalk, phenotypic plasticity, plant development.	[20]
DELLA, GRAS, GAs, Arabidopsis, rice, GID1, crosstalk,	[22]
ABA, ABA synthesis, ABA signaling, ABA response.	[35]
PYR1, RCAR, ABA transduction, SnRK2, PP2C.	[42]
ABI5, abiotic stress response, phytohormone crosstalk, ABA, brassinosteroids, GAs, jasmonates, posttranslational modification.	[81]
**Hormones transport**	
GA biosynthesis, GA action, GA transport, evolution, *Gibberella fujikuroi*.	[48]
GA, plant hormone, hormone transport and localization, GA transporters, NPF, SWEET.	[49]
ABA; guard cell; transporter; water stress.	[50]
GA metabolism, GA transport, GA gradients, cellular growth.	[51]
Plant NRT1/PTR transporters, phylogenetic relationships, substrates.	[72]
Glucosinolate, hormone, nitrate, nitrite, peptide, transporter.	[73]
NIN-like protein (NLP), nitrate, nitrate-responsive gene expression, post-translational regulation, transcription factor, nitrate signalling.	[77]
**Light regulation**	
PIF signaling, Phytochromes, plant development.	[109]
**Chromatin dynamics**	
Shoot apical meristem, quiescent center, floral homeotic gene, polycomb repression, seed development, dormancy, germination and greening.	[162]
DNA methylation, genomic imprinting, Polycomb group proteins, plants.	[309]
**Small RNAs**	
Seed germination, seed, small RNA, miRNA, stress response, seed dormancy.	[189]
microRNA, siRNA, Arabidopsis.	[202]
**Postranslational modifications**	
ABA, ABA signaling, post-translational regulation, phosphorylation, dephosphorylation, ubiquitination, 26S proteasome system.	[232]
**Dormancy**	
Environmental cueing, extinction, germination, physiological dormancy, speciation.	[2]
Seed dormancy, germination, ABA, GAs, phytohormone crosstalk.	[239]
ABA, dormancy, germination, GAs, plant hormones, preharvest sprouting, seed, cereals.	[240]
Cereal grain, cereals, pre-harvest sprouting, seed dormancy, seed germination.	[241]
Crop seed dormancy, germination, phytohormones, pre-harvest sprouting.	[242]
Dormancy, germination, endosperm weakening, DOG1, ABA signaling.	[260]

Environment, FLC, flowering time, maternal, seed coat, seed development, seed dormancy, seed germination, tannin, temperature.	[308]
Pre-dispersal, post-dispersal, predation, seed ageing, seed decay, seed defence, seed dispersal, seed dormancy, seed longevity, seed persistence.	[355]
Annual life cycle, Arabidopsis, DOG1, dormancy cycling, germination, nitrate signalling, PHYA, seed dormancy.	[356]
**Other hormones and ROS**	
NO, ABA, dormancy, germination, seed, vigor.	[138]
NO, dormancy, germination, hypocotyl elongation, reactive nitrogen species, root development, seeds.	[139]
Carbon monoxide (CO), abiotic stress, growth and development, antioxidant defense, physiological role, signaling transduction.	[177]
Ethylene, ABA; dormancy; GAs; ROS; seed germination.	[285]
Karrikins, germination, smoke, signaling, strigolactone.	[292]
ROS, Abiotic stress response, molecular regulators, phytohormones, PCD, seed germination.	[315]
ROS, seed physiology, germination, seed dormancy, signalling, embryogenesis, PCD.	[337]
ROS, dormancy, germination, hydrogen peroxide, phytohormone, priming, seed, signaling molecule.	[338]
ROS, aging seeds, antioxidant system, DNA damage, methylation.	[341]
**AR and longevity**	
Glassy state, seed, longevity, viscosity, desiccation tolerance, non-reducing sugars, LEA proteins, stability, dry state, molecular mobility, molecular density, hydrogen-bonding.	[312]
Desiccation tolerance, ABA, chlorophyll, longevity, LEA proteins, raffinose family oligosaccharides.	[313]
AR, dormancy, hormones, seed storage, oxidative reactions, seed bank, early imbibition.	[316]
AR, ABA, Arabidopsis, dormancy, endosperm, germination, seed.	[317]
Seed germination, dormancy, longevity, seed development, RNA-binding proteins, α-amanitin, proteome, translatome.	[324]
Desiccation tolerance, proteome, seed developments seed germination, seed vigor.	[336]
DNA repair, seeds, germination, priming, aging.	[344]
Anhydrobiosis, desiccation, dormancy, hormone, longevity, oxidation.	[348]
**Biomechanics**	
Endosperm, embryo, germination, seed, ABA, GAs.	[362]
Angiosperms, apoplastic ROS, biological materials, seed coat, seed development, embryo growth potential, endosperm weakening, germination, puncture force, seed biomechanics.	[364]

Table S2. List of complete gene names in alphabetical order according to their acronyms.

Abbreviation/ Gene Name	Complete Name	Gene Type
ABA2	*ABSCISIC ACID DEFICIENT 2*	Abscisic acid biosynthetic enzyme
ABCG	*ATP BINDING CASSETTE (ABC) G*	G subfamily of ABC transporter
ABF/AREB	*ABSCISIC ACID-RESPONSIVE ELEMENT (ABRE)-BINDING FACTOR*	Basic leucine zipper (bZIP) TF
ABI3	*ABSCISIC ACID INSENSITIVE 3*	B3 domain-containing TF
ABI4	*ABSCISIC ACID INSENSITIVE 4*	APETALA2/Ethylene Responsive Factor (AP2/ERF) TF

ABI5	*ABSCISIC ACID INSENSITIVE 5*	Basic leucine zipper (bZIP) TF
ACO	*1-AMINOCYCLOPROPANE-1-CARBOXYLIC ACID (ACC) OXIDASE*	Ethylene biosynthetic enzyme
AGL	*AGAMOUS-LIKE*	MCM1/Agamous/Deficiens/SRF (MADS)-box TF
AHG1	*ABA HYPERSENSITIVE GERMINATION 1*	Protein phosphatase 2C (PP2C)
AHG3	*ABA HYPERSENSITIVE GERMINATION 3*	Protein phosphatase 2C (PP2C)
AHT1	*ABA-HYPERSENSITIVE BTB/POZ PROTEIN1*	Broad-complex, Tramtrack, and Bric-à-brac/poxvirus and zinc finger (BTB/POZ)-domain-containing protein
AL	*ALFIN1-LIKE PROTEIN*	Plant homeodomain (PHD) finger protein
ALE1	*ABNORMAL LEAF-SHAPE 1*	PA domain-containing subtilase
ALN	*ALLANTOINASE*	Allantoin degradation and assimilation enzyme
AOS	*ALLENE OXIDE SYNTHASE*	JA biosynthetic enzyme
ARF	*AUXIN RESPONSE FACTOR*	ARF TF
ARR	*ARABIDOPSIS RESPONSE REGULATOR*	ARR TF
ATHB	*ARABIDOPSIS THALIANA HOMEOBOX*	Homeodomain-Leucine zipper (HD-Zip) TF
ATM	*ATAXIA TELANGIECTASIA MUTATED*	Phosphatidylinositol 3 kinase-like (PI3K) protein kinase
ATML1	*ARABIDOPSIS THALIANA MERISTEM LAYER 1*	Homeodomain-Leucine zipper (HD-Zip) TF
ATR	*ATM AND RAD3-RELATED*	Phosphatidylinositol 3 kinase-like (PI3K) protein kinase
ATS2	*ACYLTRANSFERASE 2*	Lysophosphatidic acid acyltransferase
ATXR7	*ARABIDOPSIS TRITHORAX-RELATED 7*	Histone-lysine N-methyltransferase
AUX1	*AUXIN TRANSPORTER PROTEIN 1*	Auxin influx carrier protein
BBX21	*B-BOX (BBX) CONTAINING ZINC-FINGER 21*	B-box zinc finger TF
BDG1	*BODYGUARD1*	Cutin biosynthetic hydrolase
BES1	*BR INSENSITIVE 1 (BRI1)-EMS-SUPPRESSOR 1*	Basic helix-loop-helix (bHLH) TF
BIN2	*BRASSINOSTEROID INSENSITIVE2*	Glycogen Synthase Kinase 3-like kinase
BRM	*BRAHMA*	SWITCH2/Sucrose non-fermentable2 (SWI2/SNF2) chromatin-remodeling ATPase
BZR1	*BRASSINAZOLE RESISTANT 1*	Basic helix-loop-helix (bHLH) TF
CDF4/DOF2.3	*CYCLIC DOF FACTOR 4/DNA-BINDING WITH ONE FINGER 2.3*	DNA-binding with one finger (DOF) TF
CHD3	*CHROMODOMAIN HELICASE-DNA BINDING 3*	ATP-dependent Chromodomain Helicase-DNA binding3 (CHD3) type chromatin-remodeling factor of the SWITCH/Sucrose nonfermentable (SWI/SNF) family
CHO1	*CHOTTO1*	Double APETALA2 (AP2) repeat TF
CHR	*CHROMATIN-REMODELING*	SWITCH2/Sucrose non-fermentable2 (SWI2/SNF2) ATPase
COG1/DOF1.5	*COGWHEEL1/DNA-BINDING WITH ONE FINGER 1.5*	DNA-binding with one finger (DOF) TF
COP1	*CONSTITUTIVE PHOTOMORPHOGENIC 1*	E3 ubiquitin ligase
COP10	*CONSTITUTIVE PHOTOMORPHOGENIC 10*	Ubiquitin-conjugating enzyme variant (UEV)

CRA1/CRU1	*CRUCIFERINA*	12S seed storage protein
CRL3	*CULLIN-RING E3 LIGASE3*	E3 ubiquitin ligase
CRU3	*CRUCIFERIN3*	12S seed storage protein
CTG10	*COLD TEMPERATURE GERMINATING 10*	KELCH F-Box protein
CTS	*COMATOSE*	Peroxisomal ATP-binding cassette transporter
CUL4	*CULLIN 4*	E3 ubiquitin ligase component
CYP	*CYTOCHROME P450*	Enzymes (CYP707As are monooxygenases, ABA catabolic enzymes)
D14	*DWARF14*	Strigolactone receptor
DAG1	*DOF-AFFECTING GERMINATION 1*	DNA-binding with one finger (DOF) TF
DCP5	*DECAPPING 5*	Decapping complex component
DDB1	*DET1–DAMAGED DNA BINDING PROTEIN 1*	COP10, DDB1, DET1 (CDD) complex component (regulates the activity of ubiquitin conjugating enzymes)
DET1	*DE-ETIOLATED 1*	COP10, DDB1, DET1 (CDD) complex component (regulates the activity of ubiquitin conjugating enzymes)
DOG1	*DELAY OF GERMINATION 1*	DELAY OF GERMINATION (DOG)-family protein
EBS	*EARLY BOLTING IN SHORT DAY*	Bromo-adjacent homology (BAH)-plant homeodomain (PHD)-containing protein
EFS	*EARLY FLOWERING IN SHORT DAYS*	Histone-lysine N-methyltransferase
EMF2	*EMBRYONIC FLOWER 2*	Polycomb group (PcG) protein
ERF	*ETHYLENE RESPONSE FACTORS*	APETALA2/Ethylene Responsive Factor (AP2/ERF) TFs
ETR1/RDO3	*ETHYLENE RESPONSE 1/ REDUCED DORMANCY 3*	Ethylene receptor
EXP8	*EXPANSIN-A8*	Alpha-Expansin protein
EXPA	*EXPANSIN*	Alpha-Expansin proteins
FLC	*FLOWERING LOCUS C*	MCM1/Agamous/Deficiens/SRF MADS-box protein
FT	*FLOWERING LOCUS T*	Phosphatidylethanolamine-binding protein
FUS3	*FUSCA3*	B3 domain-containing TF
FyPP	*PHYTOCHROME-ASSOCIATED SERINE/THREONINE PROTEIN PHOSPHATASE*	Type 6 serine/threonine protein phosphatase (PP6) phosphatase
GA20ox	*GA 20-OXIDASES*	GA biosynthetic enzyme
GA2ox	*GA 2-OXIDASES*	GA catabolic enzyme
GA3ox	*GA 3-OXIDASES*	GA biosynthetic enzyme
GAI	*GIBBERELLIC ACID INSENSITIVE*	DELLA protein
GARU	*GA RECEPTOR RING E3 UBIQUITIN LIGASE*	E3 ubiquitin ligase
GASA6	*GIBBERELLIC ACID-STIMULATED ARABIDOPSIS 6*	GASA peptide
GATA12	*GATA TRANSCRIPTION FACTOR 12*	GATA-type zinc finger TF
GID1	*GIBBERELLIN INSENSITIVE DWARF 1*	GA receptor
GID2	*GIBBERELLIN INSENSITIVE DWARF 2*	F-box factor
GSO	*GASSHO*	Leucine-rich repeat transmembrane-type receptor kinase
HD2B	*HISTONE DEACETYLASE2B*	Histone deacetylase
HDA	*HISTONE DEACETYLASE*	Histone deacetylase
HDAC	*HISTONE DEACETYLASE*	Histone deacetylase
HFR1	*LONG HYPOCOTYL IN FAR-RED 1*	Basic helix-loop-helix (bHLH) TF

HSI2	*HIGH-LEVEL EXPRESSION OF SUGAR-INDUCIBLE GENE 2*	B3 domain-containing TF
HUB1/RDO4	*HISTONE MONOUBIQUITINATION 1/REDUCED DORMANCY 4*	E3 ubiquitin ligase
HUB2	*HISTONE MONO-UBIQUITINATION 2*	E3 ubiquitin ligase
HY1	*HEME OXYGENASE 1*	Phytochrome chromophore biosynthetic enzyme
HY5	*LONG HYPOCOTYL 5*	Basic leucine zipper (bZIP) TF
HYH/bZIP64	*HY5-LIKE*	Basic leucine zipper (bZIP) TF
ICE1	*INDUCER OF CBF EXPRESSION 1*	Basic helix-loop-helix (bHLH) TF
JAR1	*JASMONATE RESISTANT 1*	Jasmonoyl-L-amino acid synthetase
JAZ1	*JASMONATE-ZIM-DOMAIN PROTEIN 1*	JA signaling repressor
JMJ	*JUMONJI*	Histone demethylase
KAI2/HTL	*KARRIKIN INSENSITIVE2 (KAI2)/ HYPOSENSITIVE TO LIGHT (HTL)*	α/β-fold hydrolase; karrikin receptor
KEG	*KEEP ON GOING*	E3 ligase
KRS	*KERBEROS*	Endosperm-specific cysteine-rich peptide
LACS2	*LONG-CHAIN ACYL-COA SYNTHETASE 2*	Fatty acid metabolic enzyme
LDL	*LYSINESPECIFIC DEMETHYLASE-LIKE*	Histone demethylase
LEC1	*LEAFY COTYLEDON 1*	Subunit of the nuclear transcription factor Y (NF-Y) CCAAT-binding TF
LEC2	*LEAFY COTYLEDON 2*	B3 domain-containing TF
LSM1	*SM-LIKE 1*	Component of the decapping machinery
LUH	*LEUNIG_HOMOLOG*	Groucho family co-repressor
MAP3K	*MITOGEN-ACTIVATED PROTEIN KINASE KINASE KINASE*	Mitogen-activated protein kinase
MAX2	*MORE AXILLARY GROWTH 2*	F-box protein
MFT	*MOTHER-OF-FT*	Phosphatidylethanolamine-binding protein
MPK/MAPK	*MITOGEN-ACTIVATED PROTEIN KINASE*	Mitogen-activated protein (MAP) kinase
MYB	*MYELOBLASTOSIS*	MYB TF
NAC	*NAM, ATAF1/2, AND CUC2*	NAC TF
NCED	*9-CIS-EPOXYCAROTENOID DIOXYGENASES*	ABA biosynthetic enzymes
NF-YC	*NUCLEAR FACTOR-Y C*	CCAAT-binding TF
NIA/NR	*NITRATE REDUCTASE*	Enzyme; asimilation of nitrate into ammonia
NIR1	*NITRITE REDUCTASE*	Enzyme; asimilation of nitrate into ammonia
NLP	*NIN-LIKE PROTEIN*	Nitrate response NLP family TF
NPF	*NITRATE TRANSPORTER1 (NRT1)/PEPTIDE TRANSPORTER (PTR)*	Hormone, peptide and nitrate transporter family
NPF4.6/AIT1/ NRT1.2	*NITRATE TRANSPORTER1 (NRT1)/PEPTIDE TRANSPORTER (PTR) (NPF) 4.6/ABA-IMPORTING TRANSPORTER (AIT) 1/NITRATE TRANSPORTER (NRT) 1.2*	ABA and nitrate NPF transporter
NRE	*NITRATE RESPONSE CIS-ELEMENT*	NLP binding sequence
NRPD1	*NUCLEAR RNA POLYMERASE D1*	RNA polymerase that mediates 24-nt short-interfering RNAs (siRNA) accumulation
PAF1C	*RNA POLYMERASE II-ASSOCIATED FACTOR 1 COMPLEX*	Pol II-associated platform that recruits factors required for Pol II progression (mammals)
PDF2	*PROTODERMAL FACTOR 2*	Homeodomain (HD-ZIP) TF

PER1	*1-CYSTEINE PEROXIREDOXIN 1*	Peroxiredoxin family of antioxidants
PIF1/PIL5	*PHYTOCHROME INTERACTING FACTOR 1/PHYTOCHROME-INTERACTING FACTOR 3-LIKE 5*	Basic helix-loop-helix (bHLH) TF
PIF6/PIL2	*PHYTOCHROME-INTERACTING FACTOR 6/PHYTOCHROME INTERACTING FACTOR 3-LIKE 2*	Basic helix-loop-helix (bHLH) TF
PIF8/UNE10	*PHYTOCHROME-INTERACTING FACTOR 8/UNFERTILIZED EMBRYO SAC 10*	Basic helix-loop-helix (bHLH) TF
PKL	*PICKLE*	ATP-dependent Chromodomain Helicase-DNA binding3 (CHD3) type chromatin-remodeling factor of the SWITCH/sucrose nonfermentable (SWI/SNF) family
PP1	*TYPE 1 SERINE/THREONINE PROTEIN PHOSPHATASE*	PPP (phosphoprotein phosphatase) family phosphatase
PP2A	*TYPE 2A SERINE/THREONINE PROTEIN PHOSPHATASE*	PPP (phosphoprotein phosphatase) family phosphatase
PP2C	*TYPE 2C SERINE/THREONINE PROTEIN PHOSPHATASE*	PPP (phosphoprotein phosphatase) family phosphatase
PP6	*TYPE 6 SERINE/THREONINE PROTEIN PHOSPHATASE*	PPP (phosphoprotein phosphatase) family phosphatase
PRC1	*POLYCOMB REPRESSOR COMPLEX 1*	Complex that catalyzes H2AK119ub
PRC2	*POLYCOMB REPRESSOR COMPLEX 2*	Complex that catalyzes H3K27me3
PWR	*POWERDRESS*	SANT (Swi3, Ada2, N-Cor, TFIIIB) domain-containing protein
PYR/PYL/ RCAR	*PYRABACTIN RESISTANCE 1/PYRABACTIN RESISTANCE 1–LIKE/REGULATORY COMPONENT OF ABA RECEPTOR*	ABA receptor
RAF	*RAPIDLY ACCELERATED FIBROSARCOMA (RAF)-LIKE KINASE*	Mitogen-activated protein kinase kinase kinase (MAP3K)
RAV1	*RELATED TO ABI3/VP1 1*	APETALA2/Ethylene Responsive Factor (AP2/ERF) and B3 domain-containing TF
RDO5/DOG18/ IBO	*REDUCED DORMANCY 5/DELAY OF GERMINATION 18/ IBRAHIM BEKIROVIĆ*	Type 2C serine/threonine protein phosphatase
RGA	*REPRESSOR OF GA1-3*	DELLA protein
RGL2	*RGA-LIKE2*	DELLA protein
RRP41L/SLG	*RIBOSOMAL RNA-PROCESSING PROTEIN 41-LIKE/SLOWER GROWTH*	RNase phosphorolytic (PH) domain-type protein; putative core subunit of the exosome
RSL1	*RING FINGER OF SEED LONGEVITY1*	E3 ubiquitin ligase
RSM1	*RADIALIS-LIKE SANT/MYB 1*	Myeloblastosis (MYB) TF
RVE	*REVEILLE*	Myeloblastosis (MYB)-like TF
SAG	*SENSITIVE TO ABA DURING GERMINATION*	Midasin homologue 1 (MDN1) domain-containing protein
SCF	*SKP1-CULLIN1-F-BOX*	Subclass of E3 ubiquitin ligase
SCL	*SCARECROW-LIKE*	GRAS TF
SDG8	*SET DOMAIN GROUP 8*	Histone methyltransferase
SDR4L	*SEED DORMANCY 4-LIKE*	Sdr4 family protein
SEC	*SECRET AGENT*	O-linked N-acetylglucosamine transferase
SIZ1	*SAP AND MIZ-FINGER DOMAIN-CONTAINING PROTEIN 1*	SUMO E3 ligase
SLY1	*SLEEPY1*	F-box protein

SMAX1	*SUPPRESSOR OF MAX2-1*	Heat-shock-related protein; karrikin signaling
SMR5	*SIAMESE-RELATED 5*	Cell cycle inhibitor
SNE/SLY2	*SNEEZY/SLEEPY2*	F-box protein
SNL	*SWITCH (SWI)-INDEPENDENT 3 (SIN3)-LIKE*	Histone deacetylase-binding factor
SnRK2	*SUCROSE NONFERMENTING-1 (SNF1)-RELATED PROTEIN KINASE*	Sucrose nonfermenting-1/ AMP-activated Protein Kinases (SNF1/AMPK) serine/threonine kinases
SOM	*SOMNUS*	C3H-type zinc finger TF
SPA1	*SUPPRESSOR OF PHYA-105 1*	Component of the COP1/SPA E3 ubiquitin-protein ligase
SPT	*SPATULA*	Basic helix-loop-helix (bHLH) TF
SPY	*SPINDLY*	O-fucosyltransferase
SUVH5/SET9	*SUPPRESSOR OF VARIEGATION 3-9 HOMOLOG PROTEIN 5/SET DOMAIN GROUP 9*	Histone-lysine N-methyltransferase
SWEET	*SUGARS WILL EVENTUALLY BE EXPORTED TRANSPORTERS*	Sugar and GA transporter
SWI2/SNF2	*SWITCH2/SUCROSE NON-FERMENTABLE 2*	ATP-dependent chromatin-remodeling family
TAGK2/CRK2	*CALCIUM-DEPENDENT PROTEIN KINASE (CDPK/CPK)-RELATED PROTEIN KINASE 2*	Tyrosine kinase
TAP46	*2A PHOSPHATASE-ASSOCIATED PROTEIN OF 46 KDA*	Protein phosphatase2A (PP2A)-associated protein
TFIIS/RDO2	*TRANSCRIPTION ELONGATION FACTOR TFIIS/REDUCED DORMANCY 2*	Transcription elongation factor
α-TIP	*ALPHA-TONOPLAST INTRINSIC PROTEIN*	Aquaporin water channel
TOPP4	*TYPE ONE PROTEIN PHOSPHATASE 4*	Serine/threonine protein phosphatase
TPL	*TOPLESS*	Groucho family co-repressor
TT10/LAC15	*TRANSPARENT TESTA 10/LACCASE-15*	Laccase-like enzyme
TWS1	*TWISTED SEED 1*	Small protein
VCS	*VARICOSE*	Decapping complex component
δ-VPE	*DELTA VACUOLAR PROCESSING ENZYME*	Caspase-1-like vacuolar processing enzyme
VQ	*FXXXVQXXTG (VQ) MOTIF-CONTAINING PROTEIN*	VQ family protein
WRKY	*WRKY DNA-BINDING PROTEIN*	WRKY TF
XRN4/AIN1/EIN5	*EXORIBONUCLEASE 4/ACC INSENSITIVE 1/ETHYLENE INSENSITIVE 5*	5′-3′ exoribonuclease
XTH31	*XYLOGLUCAN ENDOTRANSGLYCOSYLASES/ HYDROLASES 31*	Xyloglucan endohydrolase and xyloglucan endotransglucosylase
XYL1/TRG1	*ALPHA-XYLOSIDASE 1/ THERMOINHIBITION RESISTANT GERMINATION 1*	alpha-l-arabinofuranosidase/beta-d-xylosidase
ZOU/RGE1	*ZHOUPI/RETARDED GROWTH OF EMBRYO 1*	Basic helix-loop-helix (bHLH) TF
ZRF	*ZUOTIN-RELATED FACTOR*	H2Aub1 deubiquitination
